# UNDERSTANDING AND RESPONDING TO THE NEEDS OF STUDENT CAREGIVERS THROUGH THE AGE-FRIENDLY UNIVERSITY LENS

**DOI:** 10.1093/geroni/igac059.1409

**Published:** 2022-12-20

**Authors:** Katherina Terhune, Allyson Graf, Kate Wade, Faith Greer

**Affiliations:** Northern Kentucky University, Lexington, Kentucky, United States; Northern Kentucky University, Florence, Kentucky, United States; Northern Kentucky University, Highland Heights, Kentucky, United States; Northern Kentucky University, Highland Heights, Kentucky, United States

## Abstract

Caregiving has steadily increased over the years, with approximately 44 million individuals in the U.S. providing over 37 billion hours of unpaid, informal care for adult family members and friends with chronic illnesses or conditions that impact their functionality and daily activities. While much caregiving research has been completed over the years, research on student caregivers is scant, and less is known about the experiences and the needs of students in caregiving roles. This presentation examines data from a campus-wide survey to assess the prevalence and impact of unpaid caregiving on students. Utilizing the Age-Friendly University framework, this study aims to identify sources of unmet caregiving need, as well as community and campus connections to better support students in caregiving roles. Recommendations for assessing student caregiver needs and ways to generate and enhance caregiver support through the AFU lens will be highlighted.

